# Heat-related mortality prediction using low-frequency climate oscillation indices: Case studies of the cities of Montréal and Québec, Canada

**DOI:** 10.1097/EE9.0000000000000206

**Published:** 2022-03-31

**Authors:** Pierre Masselot, Taha B.M.J. Ouarda, Christian Charron, Céline Campagna, Éric Lavigne, André St-Hilaire, Fateh Chebana, Pierre Valois, Pierre Gosselin

**Affiliations:** a Department of Public Health, Environments and Society, London School of Hygiene and Tropical Medicine (LSHTM), London, United Kingdom; b Institut National de la Recherche Scientifique, INRS, Centre Eau Terre Environnement, Québec, Canada; c Institut National de Santé Publique du Québec, INSPQ, Québec, Canada; d Air Health Science Division, Health Canada, Ottawa, Canada; e School of Epidemiology and Public Health, University of Ottawa, Ottawa, Canada; f Observatoire Québécois de l’Adaptation aux Changements Climatiques, Faculté des Sciences de L’éducation, Université Laval, Québec, Canada; g Ouranos, Montréal, Canada

**Keywords:** Heat, Mortality, Climate indices, Atlantic multi-decadal oscillation, Functional linear regression, Distributed lag nonlinear models

## Abstract

Heat-related mortality is an increasingly important public health burden that is expected to worsen with climate change. In addition to long-term trends, there are also interannual variations in heat-related mortality that are of interest for efficient planning of health services. Large-scale climate patterns have an important influence on summer weather and therefore constitute important tools to understand and predict the variations in heat-related mortality.

**Methods:**

In this article, we propose to model summer heat-related mortality using seven climate indices through a two-stage analysis using data covering the period 1981–2018 in two metropolitan areas of the province of Québec (Canada): Montréal and Québec. In the first stage, heat attributable fractions are estimated through a time series regression design and distributed lag nonlinear specification. We consider different definitions of heat. In the second stage, estimated attributable fractions are predicted using climate index curves through a functional linear regression model.

**Results:**

Results indicate that the Atlantic Multidecadal Oscillation is the best predictor of heat-related mortality in both Montréal and Québec and that it can predict up to 20% of the interannual variability.

**Conclusion:**

We found evidence that one climate index is predictive of summer heat-related mortality. More research is needed with longer time series and in different spatial contexts. The proposed analysis and the results may nonetheless help public health authorities plan for future mortality related to summer heat.

**What this study adds**

This study shows that the interannual variability of heat-related mortality might be partly driven by large-scale climatic teleconnections, specifically the Atlantic Multidecadal Oscillation in the case of the province of Quebec. To do so, we provide a rigorous methodology that uses recent relevant statistical development, in particular from functional data analysis that allows the consideration of several climate indices and lags. As far as the authors are aware, this study is the first of its kind, and we think it is valuable both for its informative results and its methodology.

## Introduction

Heat-related mortality is an important public health burden in summer^1^ and is expected to increase with climate change.^2,3^ To inform adaptation policies, studies have focused on modeling the evolution of mortality attributable to heat^4^ or relating the vulnerabilities across the world to socio-economic drivers.^5,6^ However, in addition to the long-term trend and to spatial variations, there is important interannual variability in summer heat and thus in heat-related mortality.^7^ It is therefore of interest to model and predict these inter-annual changes to inform short-term action plans.

Temperature is widely driven by climatic teleconnections which contribute to define large-scale climatic variations. For instance, long-term cycles in sea surface temperature in the Atlantic Ocean are strongly linked to the North American and European summer climates. Climatic teleconnections and large-scale patterns are summarized by different climate indices that represent diverse variations across the globe, such as the Southern Oscillation Index (SOI) measuring the so-called El-Niño phenomenon or the Atlantic Multidecadal Oscillation (AMO) to represent variations of sea surface temperatures in the Atlantic.^8^ These indices can then be used to predict local climate characteristics like the frequency and intensity of heatwaves.^9–11^ In addition to heat waves, these indices also predict variations of other weather variables such as precipitations,^12–14^ thus potentially affecting the vulnerability of populations to extreme meteorological events.

Given the strong links between climate indices and local weather, they are expected to be instrumental for the prediction of heat-related mortality. As accurate forecasts of climate indices several months in advance are now a prospect,^15^ they can be used to provide heat-related health forecasts months in advance. This strategy is often considered for infectious diseases that are particularly climate-sensitive,^16,17^ but less so for heat-related mortality.

One limiting factor of integrating large-scale climate information is the difficult statistical problem of predicting heat-related mortality by using potentially numerous interrelated and lagged climate indices. In the only contribution attempting it, Majeed et al.^18^ focused on two indices for the United-Stated: the ENSO phenomenon and the AMO. They used mortality causes defined a priori for heat-related mortality and performed a spectral analysis of time series. However, such an approach does not necessarily represent accurately the mortality actually caused by heat, nor allow disentangling the different climate indices that could impact summer heat.

We propose here a two-stage statistical analysis that consists in first estimating heat-related mortality for each year, and second to model annual heat-related mortality using values from climate indices. Similar two-stage methodologies have been employed to study adaptation to heat and cold^4^ as well as the differential vulnerability patterns over the world.^5^ The first stage uses state-of-the-art epidemiological models to estimate the part of mortality that can be attributed to heat. The second stage considers functional regression to efficiently integrate the whole information provided by several months of climate indices and predict the magnitude of upcoming heat-related mortality.^19^ Functional regression takes continuous functions as inputs and is thus particularly adapted to time-indexed processes. In particular, we use the functional linear array model recently proposed by Brockhaus et al.^20^ to manage the high-dimensionality of the model. Its boosting-based algorithm is efficient both for prediction and for variable selection.^21^ This allows to select the most predictive indices as well as understand how their variations can help predict heat-related mortality.

The objective of the present article is to relate mortality attributable to heat with large-scale climate indices using the two-stage methodology sketched above. As climate indices, we consider both the AMO and the SOI measuring the ENSO phenomenon, as well as five additional potentially predictive climate indices: the Arctic Oscillation (AO), the North Atlantic Oscillation (NAO), the Oceanic Niño Index (ONI), the Pacific Decadal Oscillation (PDO), and the Pacific North American Index (PNA). The obtained model can then be used to predict the magnitude of upcoming heat-related mortality and inform heat early warning systems several months in advance.

## Methods

### Data

**Figure 1.**
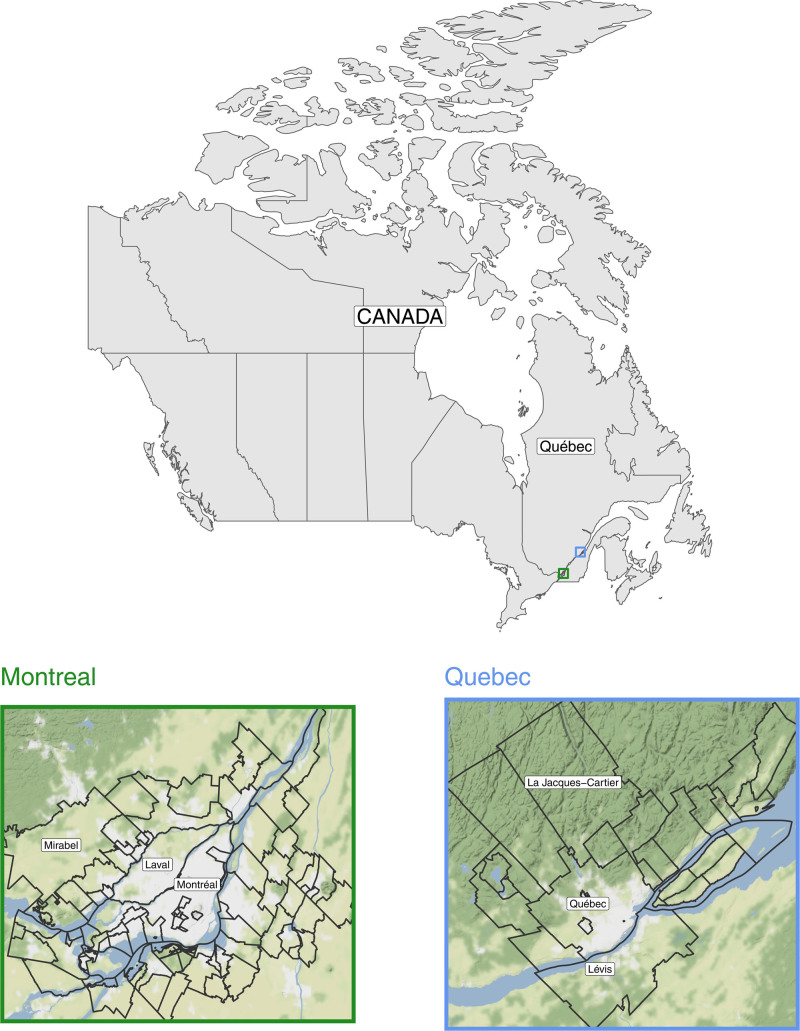
Map of the considered metropolitan areas: Montréal (green) and Québec (blue).

We consider data from two metropolitan areas (MA) in the province of Québec, Canada: Montréal and Québec. These communities correspond to the extended urban areas that include neighbor municipalities such as Laval for Montréal and Lévis for Québec as shown in Figure 1. For both MAs, we consider daily mean temperature and all-cause mortality counts as well as monthly climate indices over the period 1981–2018.

Daily time series are herein restricted to the five warmest months of the year which are May to September. All-cause mortality counts are provided for each MA by the National Institute of Public Health of Québec (*Institut national de santé publique du Québec*). Mean temperature is constructed as the average between minimum and maximum temperature, both extracted from the Daymet database (daymet.ornl.gov). Daymet is a gridded weather database with a resolution of 1 km over North America.^22^ It is bias-corrected and validated using ground observations and its high resolution allows for an accurate representation of exposure to temperature.^23^ Daily time series of mean temperature are obtained by averaging over all grid points that cover each MA.

Monthly values of the seven climate indices given above are obtained from the National Oceanic and Atmospheric Administration (NOAA) Earth System Research Laboratory (https://psl.noaa.gov/data/climateindices/list/). These seven indices are suspected to influence the climate of Southern Québec to different degrees.^24^ Each index is detailed in Appendix A; http://links.lww.com/EE/A184.

### Statistical analysis

To study the ability of climate indices to predict heat-related mortality, we consider a two-stage approach applied to each MA. In the first stage, we estimate annual heat-attributable fractions (AF) of mortality through a distributed lag nonlinear model. In the second, AF estimates are considered as the response in a functional regression model with climate index curves as predictors.

#### Estimation of heat attributable fractions

To estimate heat AF in the first stage, we apply a time-varying distributed lag nonlinear model. It consists in a quasi-Poisson regression with a cross-basis to represent the nonlinear and delayed effect of temperature on all-cause mortality.^25^ The cross-basis is parameterized following previous studies on heat-related mortality,^26^ i.e., through a quadratic B-spline with knots at percentiles 50% and 90% on the temperature dimension, and a cubic natural spline with two interior knots uniformly distributed on the log scale on the lag dimension, up to a maximum lag of 10 days. To allow the exposure-response function to evolve along the study period, and thus account for potential long-term adaptation, we add an interaction term between the cross-basis and the date as described by Gasparrini et al.^26^ The model also includes a day-of-week factor to control for weekend effects, a natural spline component of year with one degree of freedom per decade to control for a long-term trend and a natural spline of day-of-season with four degrees of freedom to control for seasonality.

The fitted model is then used to compute daily AFs for heat using classical formulas.^27^ The daily AFs are then aggregated by years including only heat days, defined as days for which mean temperature exceeds a specific threshold. Several such thresholds are considered to test whether overall heat or only the most extreme events can be predicted by climate indices (see examples in Appendix B; http://links.lww.com/EE/A184).^28^ First, we consider the minimum mortality temperature (MMT) for each year, i.e., we retrieve the overall cumulative exposure-response function^29^ at the first of July of each year and extract the temperature at which the risk is the lowest, with the constraint that it is between its 10^th^ and 90th percentile.^30^ We also consider percentiles 95%, 97.5%, and 99% of summer temperature, computed on the whole period. We focus on AF instead of attributable number (AN) as it is less prone to confounding by population.

Uncertainty of reported MMTs and AFs is assessed by 95% empirical confidence intervals (eCI) that simulate new first-stage coefficients from a multivariate normal distribution and computes the MMT and AFs for each set of simulated coefficients.^27^

#### Prediction by climate indices

For each MA and each heat threshold, estimated AFs are predicted by climate index curves through a functional regression model in which the response is scalar and the predictors are functional.^31^ This model estimates the effect of each considered month of the climate indices on AF through the following model:


$$AF_{i}=\overset{7}{\underset{j=1}{\sum }}\overset{16}{\underset{0}{\int }}\beta_{j}(l)x_{ij}\left(l\right)\;\;dl+s\left(i\right)+\varepsilon_{i},$$ (1)

where $AF_{i}$ is the heat AF of year *i*, $x_{ij}\left(l\right)$ is the curve of the $j^{th}$ climate index (j=1,  …  ,7) for year *i* with the associated functional coefficient $\beta_{j}(l)$. This coefficient indicates the change in AF induced by $x_{.j}\left(l\right)$ at each lag *l*. As the impact of climate indices on the local climate can be delayed by up to two years,^12^ in this study $x_{.j}\left(l\right)$ spans a period of 16 months from January of year i−1 to May of year *i*. Finally, $s\left(i\right)$ is a smooth component of the year expanded by P-splines, i.e., a dense cubic B-spline with 20 interior knots that is penalized to control its smoothness.^32^ The last component $\varepsilon_{i}$ represents the residuals.

To estimate model (1), we consider the functional linear array model representation with associated boosting optimization developed by Brockhaus al.^20^ In the linear array representation, the functional variables $\beta_{j}(l)$ and $x_{ij}\left(l\right)$ are expanded by spline tensor products to map discrete data to the continuous domain of l. Using this expansion, the model is then estimated through gradient boosting, which iteratively constructs the model by adding a very simple component of a single variable at each step to reduce the residual sum of squares.^21^ This algorithm served the double purpose of optimization as the residual sum of squares is iteratively reduced while the model gains in complexity, and variable selection as only the most strongly predictive explanatory variables are added in the model. The number of iterations is chosen by 10-fold cross-validation. More details on the estimation procedure and chosen number of iterations are given in Appendix C; http://links.lww.com/EE/A184.

To evaluate the predictive power of each model, we compute the *R*^2^ through 10-fold cross-validation, a procedure that does not favor more complex models, in contrast with its training sample counterpart.^33^ As an additional sensitivity analysis, we apply a model that adds the average summer temperature as an additional term in model (1) for each outcome and each location. Results of this sensitivity analysis are reported in Appendix D (http://links.lww.com/EE/A184) and show consistency with the main results reported below.

The analysis is performed using R 4.1.0^34^ with the addition of packages dlnm^35^ for first-stage heat-related mortality estimation and FDboost^36^ for the second-stage functional regression.

## Results

Summary statistics of deaths and temperature are reported in Table 1 for each MA. The study covers more than 400,000 deaths, with five times more deaths reported in Montréal than Québec. Montréal also shows a higher average temperature than Québec, being on average 3°C warmer. MMTs are also much warmer in Montréal, ranging from 16.3°C (95%eCI: 13.8–18.8) in 1981 to 19.1°C (95%eCI: 11.2–23.2) in 2018, compared to MMTs of 8.0°C (95%eCI: 8.0–20.0) in 1981 that increased to 14.3°C (95%eCI: 10.3–16.3) in 2018 for Québec.

**Table 1.** Total number of deaths and mean temperature summary by area.

Area	Deaths	Daily mean temperature (mean) (range)	Annual minimum mortality temperature (MMT) range (95%eCI)
Montréal	354 764	17.6 (1.3–29.2)	16.3 (13.8–18.8) 19.1 (11.2–23.2)
Québec	73 199	14.4 (−1.4–26.6)	8.0 (8.0–20.0) 14.3 (10.3–16.3)

**Figure 2.**
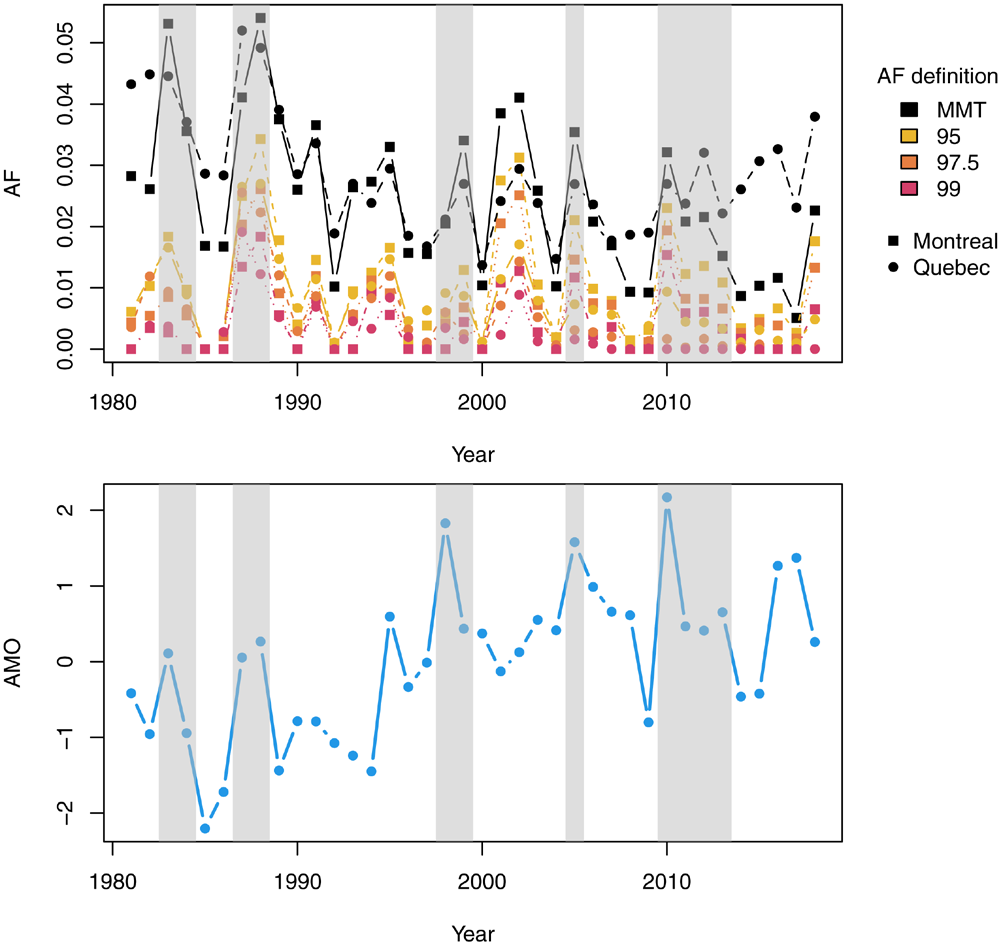
Estimated attributable fractions (AF) for each year, area and percentile (top), and the average of the same year March to May AMO (bottom). Grey areas indicate periods when both AF and AMO peak.

Figure 2 shows AF estimated for both MAs and different percentiles defining heat-related mortality, with detailed numbers and eCI in Tables S1 and S2; http://links.lww.com/EE/A184. Related results such as dose-response curves and summary statistics are reported in Appendix C; http://links.lww.com/EE/A184. On average 2.42% (95%eCI: 2.02–3.48) of mortality is attributed to heat above the MMT in Montréal and 2.84% (95%eCI: 1.32–5.88) in Québec, and the AF decreases with the percentile considered with an average of 0.40% (95%eCI: 0.36–0.45) in Montréal and 0.24% (95%eCI: 0.15–0.34) in Québec for heat defined with the 99th percentile. In both MAs, AF widely varies from year to year with a downward trend visible for MMT-related AFs. This downward trend is accompanied by an increase of MMT in recent years (see Figure S2; http://links.lww.com/EE/A184 in Appendix B) and suggests a possible diminution of the overall vulnerability to heat, consistently with previous studies.^4,26^ Figure 2 also highlights years with peaks for both mortality attributed to heat and AMO values such as 1983, 1988, 1999, 2005, and 2010.

**Figure 3.**
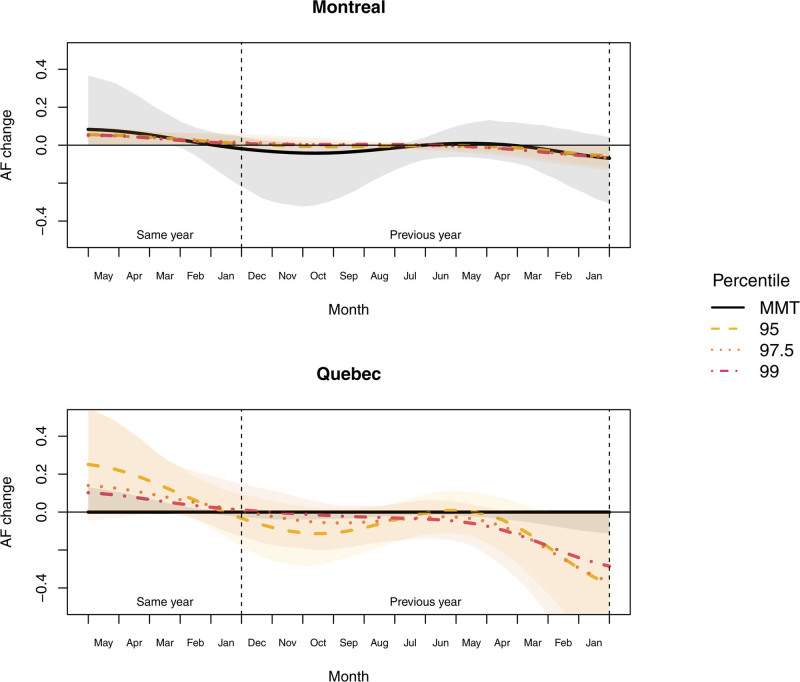
Scaled functional coefficients β^j. with their 95% confidence intervals in Montréal and Québec for all heat definitions. The curves span 16 months lag from the beginning of summer (lag 0) on the left to January of the previous year on the right (lag 16).

For both MAs and all heat definitions, results from the second-stage functional regression model are similar. Details on intermediate results are shown in Appendix D; http://links.lww.com/EE/A184. In all cases, AMO was the single climate index selected, except for Québec and the MMT-related AF for which the model selected only the time component due to the important trend. Figure 3 shows the estimated functional coefficients β^j. from Equation 1. In all cases, the coefficient is positive for lags 0 to 5, i.e., in the same year, winter and spring, and are close to zero afterward, with negative values during winter of the previous year. Therefore, a low AMO value in winter and spring is often followed by an important AMO value in winter and spring of the following year and then higher than usual heat-related mortality in summer. This is visually confirmed in Figure 2 which shows a similar pattern between AFs and the average of the same year January to May AMO. Figure 3 also indicates a higher amplitude of scaled β^j. for when AFs are defined with the MMT in Montréal. This suggests a link between the AMO and the overall heat-related mortality, not only with the most extreme heat waves.

Table 2 indicates the cross-validated *R*^2^ statistic for each model. The prediction of AF is better in Québec than in Montréal with *R*^2^ values decreasing from 21.8% for MMT-related AF to 2.0 % for the 99th percentile AF for Montréal and decreasing from 51.9% to 14.5% for Québec. These numbers are higher in Québec because of the important decreasing trends of AF that are mainly predicted by the time component while the AMO predicts the year-to-year variations.

**Table 2.** Cross-validated *R*^2^ (standard deviation) expressed in percentage for each second-stage model.

Percentile defining heat	Montréal	Québec
MMT	20.1 (1.9)	51.8 (0.8)
95th	1.8 (0.4)	21.5 (2.7)
97.5th	2.0 (0.5)	26.0 (4.7)
99th	2.0 (0.4)	13.2 (1.7)

## Discussion

In the present work, we predicted summer heat-related mortality in two MAs of the province of Québec using climate indices. Results indicated that a non-negligible part of heat-related mortality can be well predicted by the AMO index, especially its values in the late winter and spring of the same year. Predictions are also better for the whole heat-related AF than for the most extreme cases. These results suggest that monitoring the AMO is relevant for public health authorities in the province of Québec to anticipate and adjust emergency preparedness for heat-related mortality on an annual basis.

The AMO index measures north-Atlantic sea surface temperatures, which shows an important impact on the North American weather.^37,38^ For instance, positive (warmer) AMO phases are associated with a decrease in rainfalls as well as an increase in droughts in the United States.^39^ In the province of Québec, AMO has been associated with generally hotter summers,^24^ as well as longer and warmer heat spells.^11^ Therefore, it is natural that AMO is partly predictive of heat-related mortality.

AMO is predictive of heat-related mortality in both Montréal and Québec, despite strong geographical and adaptation trends disparities between the two cities. Indeed, the MMT differs by approximately 6°C which confirms previous works indicating higher risks for similar temperature in Québec compared to Montréal.^40^ A variety of factors can explain this difference, including generally lower temperatures in Québec, a less pronounced urban heat island effect being a more spread and less dense city, as well as closer proximity to the Atlantic Ocean.^41^ First-stage results also indicate a decreasing heat-related risk during the study period, which is in line with previous results in Québec^42^ and across the world.^26^

Results suggest that AMO is not only predictive of extreme heat waves-related mortality but of the whole heat-related mortality. Indeed, in Québec, the whole temperature distribution seems correlated to climate teleconnections.^24^ Besides, the number of extreme events is still low over less than forty years of data, meaningless data to properly train predictive models. Therefore, further research is needed to be able to predict the deadliest heatwave events.

The main strength of this work is the statistical methodology that uses state-of-the-art approaches in both stages. First, heat AFs are estimated by time-varying distributed lag nonlinear models, a methodology recently used to highlight long-term adaption phenomena across the world.^4,26^ Second, the associations between climate indices and heat AF are estimated through functional regression, an increasingly popular methodology in many fields. It has been considered a few times in epidemiology,^43^ including environmental epidemiology,^44,45^ as well as in climatic studies to forecast temperature.^46^ Its efficient use of intrinsically continuous variables allows the inclusion of monthly records of climate indices, without the need to remove the highest frequencies and select specific months beforehand as was done before.^18,24^

The present work shows several shortcomings, due to the complexity of climate and health relationships. The number of years considered is still low to fully apprehend the impact of teleconnections on heat-related health, especially for the most extreme events. Indeed, many teleconnections represented by the considered climate indices present low-frequency patterns that cannot be fully captured by only 38 years of data. For instance, one of the main periodicities of AMO is several decades long and this mode is not captured by our dataset, although the smaller scale oscillations of AMO predict well heat-related mortality. In addition, the second stage model is fitted with only 38 individual records, preventing the fitting of very complex models with several indices and potential interactions. Therefore, studies relating large-scale climate patterns and heat may gain strength as data accumulates in the upcoming years.

The second shortcoming, as suggested by the prediction criteria computed here, is that many factors that drive the variation in heat-related mortality could not be accounted for in this study. These include the prevalence of air conditioning^6^ or the introduction of early heat-health warnings in the province of Québec.^47,48^ In addition, the already complex time-varying DLNM fitted in this work does not capture short-term variations in the heat-related risk such as seasonal adaptation, that could alter the true AF.^49^

More research is needed to better understand how climatic teleconnections can predict heat-related mortality and how to efficiently integrate such information in heat-wave warning systems. This includes studies with much longer time series of mortality and temperature. As the present work focuses on the province of Québec, it is important to replicate it in locations with different climates in which other climatic indices will be better predictors of heat-related mortality. In addition, the present work focuses on all-cause mortality and specific mortality causes could be best predicted by teleconnections. Finally, the present work could also be extended to cold-related mortality, as winter extreme cold and precipitations are also widely linked to the different climatic patterns.^50^

The main conclusion of the present work is the possibility to predict summer heat-related mortality using climate indices representing large-scale teleconnections. For the specific case of the Québec province, it was found that the best index for this task is the AMO. This information can help inform public health seasonal heat action plans.

## ACKNOWLEDGMENTS

### Conflicts of interest statement

The authors declare that they have no conflicts of interest with regard to the content of this report.

## Supplementary Material


