# Supplementary material for: Heat-related mortality prediction using low-frequency climate oscillation indices: Case studies of the cities of Montréal and Québec, Canada
Source: Environ Epidemiol. 2022 Mar 31;6(2):e206. doi: 10.1097/EE9.0000000000000206 (PMC9005246; doi:10.1097/EE9.0000000000000206)
Supplement: Supplementary file 1 [file ee9-6-e206-s001.docx]

Heat-related mortality prediction using low-frequency climate oscillation indices; Case studies of the cities of Montreal and Quebec, Canada

**Supplementary information**

# Climate indices

This appendix details climate indices below and Figure S1 shows their values for the study period.


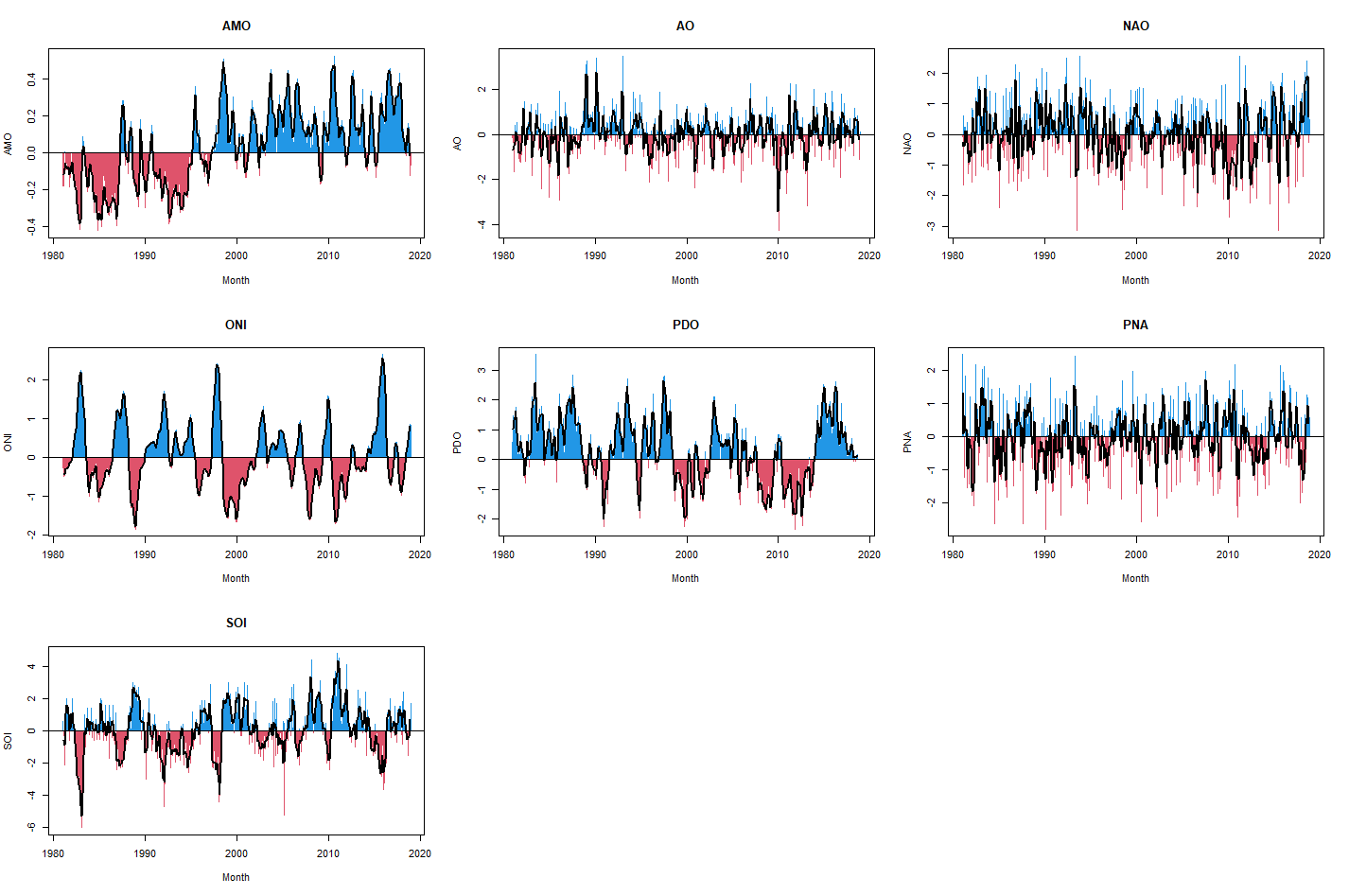


Figure S1: Monthly climate indices values from 1981 to 2018 with blue corresponding to positive phases and red to negative phases. The black line represents the 3-month moving average on each index.

## A.1. AMO

The Atlantic Multi-decadal Oscillation represents the variations of sea-surface temperatures (SST) in North Atlantic. It is computed as the average of anomalies of SST across the whole basin and represents a simple indicator of climate variability in the North Atlantic area. It has been linked to variations of temperatures both in North America (Assani et al., 2019) and Europe (Ghosh et al., 2017).

## A.2. AO

The Arctic Oscillation represents sea-level pressure anomalies in the Artic and is an indicator of the degree to which air from the Arctic penetrates into lower latitudes. When it is positive, lower cold air migrates to middle latitude and the climate is warmer. Important negative phases have in particular a large influence on winter weather in Europe and North-America (Thompson and Wallace, 1998).

## A.3. NAO

Strongly related to the AO, the North Atlantic Oscillation represents the strength and direction of winds and storms across the North Atlantic. It is measured as the difference in atmospheric pressure at sea level between Iceland and the Azores. It has notably been linked to unusually high snowfalls in the United-States and Europe (Seager et al., 2010).

## A.4. ONI

The Oceanic Nino Index represents the so-called El-Nino over southern Pacific Ocean. It is computed through SSTs over a region in the southern Pacific Ocean. As El-Nino is one of the major climate phenomenon of the planet, it has a global influence on climate, including in North America (Gershunov, 1998). As such, it has been linked to heat-related mortality in the United-States (Majeed et al., 2020).

## A.5. PDO

The Pacific Decadal Oscillation measures SST anomalies across the northern Pacific Ocean in a similar way to ONI, although with a longer time scale. It has important effects on the weather and water resources in North America (Mantua and Hare, 2002), including the eastern part (McCabe et al., 2004).

## A.6. PNA

The Pacific-North American teleconnection pattern represents the atmospheric circulation pattern over the North American continent. It is particularly linked to above and below average temperature in Canada and United-States.

## A.7. SOI

The Southern Oscillation Index represents variations in the monthly fluctuations in the air pressure difference between Tahiti and Darwin, and is a proxy for the so-called El-Nino episodes. This index is thus associated with weather and temperature across the whole globe.

# First stage modelling results


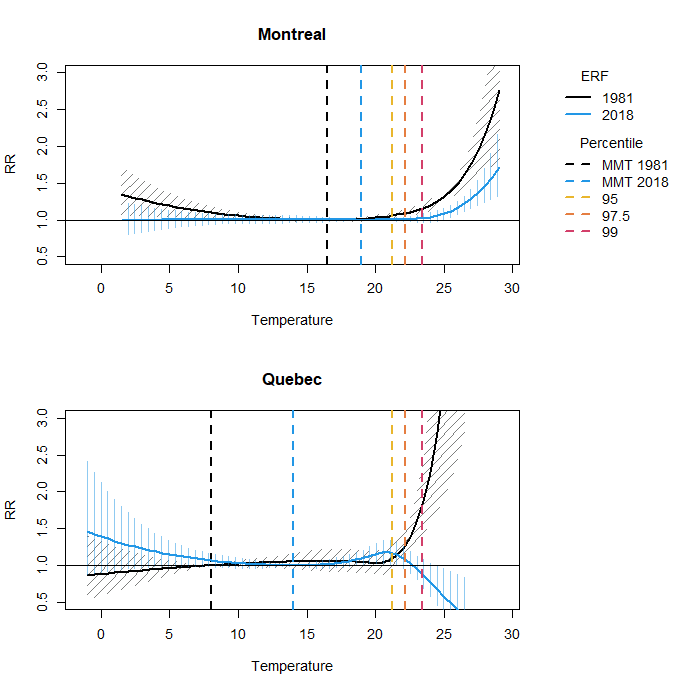


Figure S2: First-stage overall cumulative exposure response function (ERF) in Montreal and Quebec along with the percentiles used to define heat-related mortality.

Table S1: Estimated attributable fraction (AF, in percentage) for each definition in Montreal with empirical confidence intervals (eCI) within brackets.

| **Year** | **MMT** | **95^th^ percentile** | **97.5^th^ percentile** | **99^th^ percentile** |
| --- | --- | --- | --- | --- |
| 1981 | 2.8 (1.9 - 4.9) | 0.6 (0.5 - 0.7) | 0.4 (0.4 - 0.5) | 0.0 (0.0 - 0.0) |
| 1982 | 2.6 (1.9 - 4.3) | 1.0 (0.9 - 1.2) | 0.5 (0.4 - 0.6) | 0.3 (0.3 - 0.4) |
| 1983 | 5.3 (3.9 - 7.1) | 1.8 (1.5 - 2.2) | 0.9 (0.7 - 1.0) | 0.3 (0.2 - 0.3) |
| 1984 | 3.6 (2.6 - 5.2) | 1.0 (0.8 - 1.1) | 0.6 (0.5 - 0.6) | 0.0 (0.0 - 0.0) |
| 1985 | 1.7 (1.0 - 3.3) | 0.0 (0.0 - 0.0) | 0.0 (0.0 - 0.0) | 0.0 (0.0 - 0.0) |
| 1986 | 1.7 (1.1 - 3.0) | 0.2 (0.2 - 0.2) | 0.2 (0.2 - 0.2) | 0.0 (0.0 - 0.0) |
| 1987 | 4.1 (3.4 - 5.6) | 2.5 (2.2 - 2.8) | 2.0 (1.7 - 2.3) | 1.3 (1.1 - 1.6) |
| 1988 | 5.4 (4.6 - 6.8) | 3.4 (3.0 - 3.9) | 2.6 (2.2 - 3.0) | 1.8 (1.5 - 2.1) |
| 1989 | 3.8 (3.0 - 5.3) | 1.8 (1.5 - 2.0) | 0.9 (0.8 - 1.0) | 0.6 (0.5 - 0.6) |
| 1990 | 2.6 (1.9 - 3.9) | 0.4 (0.3 - 0.5) | 0.0 (0.0 - 0.0) | 0.0 (0.0 - 0.0) |
| 1991 | 3.7 (2.9 - 5.1) | 1.5 (1.3 - 1.6) | 1.2 (1.0 - 1.4) | 0.8 (0.7 - 0.9) |
| 1992 | 1.0 (0.6 - 2.3) | 0.1 (0.1 - 0.1) | 0.0 (0.0 - 0.0) | 0.0 (0.0 - 0.0) |
| 1993 | 2.6 (2.0 - 3.9) | 0.9 (0.8 - 1.1) | 0.6 (0.5 - 0.6) | 0.0 (0.0 - 0.0) |
| 1994 | 2.7 (2.2 - 3.8) | 1.2 (1.1 - 1.4) | 1.1 (1.0 - 1.3) | 0.9 (0.8 - 1.1) |
| 1995 | 3.3 (2.7 - 4.4) | 1.7 (1.5 - 1.9) | 0.9 (0.8 - 1.0) | 0.6 (0.5 - 0.6) |
| 1996 | 1.6 (1.1 - 2.9) | 0.1 (0.1 - 0.1) | 0.0 (0.0 - 0.0) | 0.0 (0.0 - 0.0) |
| 1997 | 1.6 (1.1 - 2.5) | 0.4 (0.3 - 0.5) | 0.0 (0.0 - 0.0) | 0.0 (0.0 - 0.0) |
| 1998 | 2.0 (1.5 - 3.2) | 0.6 (0.5 - 0.7) | 0.4 (0.4 - 0.5) | 0.0 (0.0 - 0.0) |
| 1999 | 3.4 (2.7 - 4.6) | 1.3 (1.1 - 1.5) | 0.7 (0.6 - 0.8) | 0.4 (0.4 - 0.5) |
| 2000 | 1.0 (0.7 - 2.0) | 0.0 (0.0 - 0.0) | 0.0 (0.0 - 0.0) | 0.0 (0.0 - 0.0) |
| 2001 | 3.8 (3.3 - 4.8) | 2.8 (2.4 - 3.1) | 2.1 (1.8 - 2.3) | 1.1 (0.9 - 1.2) |
| 2002 | 4.1 (3.6 - 5.0) | 3.1 (2.8 - 3.5) | 2.5 (2.2 - 2.8) | 1.3 (1.1 - 1.4) |
| 2003 | 2.6 (2.0 - 3.7) | 1.1 (0.9 - 1.2) | 0.7 (0.6 - 0.8) | 0.3 (0.2 - 0.3) |
| 2004 | 1.0 (0.7 - 2.1) | 0.2 (0.2 - 0.2) | 0.0 (0.0 - 0.0) | 0.0 (0.0 - 0.0) |
| 2005 | 3.5 (2.8 - 4.8) | 2.1 (1.8 - 2.4) | 1.5 (1.3 - 1.7) | 1.2 (1.0 - 1.3) |
| 2006 | 2.1 (1.5 - 3.3) | 1.0 (0.8 - 1.2) | 0.7 (0.7 - 0.9) | 0.2 (0.2 - 0.3) |
| 2007 | 1.7 (1.2 - 2.9) | 0.8 (0.7 - 0.9) | 0.7 (0.6 - 0.8) | 0.4 (0.3 - 0.4) |
| 2008 | 0.9 (0.5 - 2.4) | 0.1 (0.1 - 0.2) | 0.0 (0.0 - 0.0) | 0.0 (0.0 - 0.0) |
| 2009 | 0.9 (0.5 - 2.2) | 0.3 (0.2 - 0.4) | 0.1 (0.1 - 0.1) | 0.0 (0.0 - 0.0) |
| 2010 | 3.2 (2.5 - 4.8) | 2.3 (1.9 - 2.7) | 1.9 (1.6 - 2.3) | 1.5 (1.3 - 1.8) |
| 2011 | 2.1 (1.4 - 3.9) | 1.2 (1.0 - 1.5) | 0.8 (0.7 - 1.0) | 0.6 (0.5 - 0.7) |
| 2012 | 2.2 (1.3 - 4.2) | 1.4 (1.1 - 1.8) | 0.8 (0.7 - 1.0) | 0.6 (0.5 - 0.7) |
| 2013 | 1.5 (1.0 - 3.4) | 1.1 (0.8 - 1.5) | 0.7 (0.5 - 0.8) | 0.3 (0.3 - 0.4) |
| 2014 | 0.9 (0.4 - 3.1) | 0.3 (0.3 - 0.4) | 0.3 (0.2 - 0.3) | 0.2 (0.1 - 0.2) |
| 2015 | 1.0 (0.4 - 3.7) | 0.5 (0.3 - 0.7) | 0.4 (0.3 - 0.6) | 0.0 (0.0 - 0.0) |
| 2016 | 1.2 (0.5 - 4.0) | 0.7 (0.4 - 1.1) | 0.4 (0.3 - 0.5) | 0.0 (0.0 - 0.0) |
| 2017 | 0.5 (0.2 - 3.2) | 0.3 (0.2 - 0.4) | 0.2 (0.1 - 0.2) | 0.0 (0.0 - 0.0) |
| 2018 | 2.3 (1.5 - 5.4) | 1.8 (1.2 - 2.5) | 1.3 (0.9 - 1.8) | 0.6 (0.4 - 0.9) |

Table S2: Estimated attributable fraction (AF, in percentage) for each definition in Quebec with empirical confidence intervals (eCI) within brackets.

| **Year** | **MMT** | **95^th^ percentile** | **97.5^th^ percentile** | **99^th^ percentile** |
| --- | --- | --- | --- | --- |
| 1981 | 4.3 (0.5 - 11.9) | 0.6 (0.4 - 0.9) | 0.4 (0.2 - 0.5) | 0.0 (0.0 - 0.0) |
| 1982 | 4.5 (0.9 - 10.9) | 1.2 (0.8 - 1.6) | 1.2 (0.8 - 1.6) | 0.4 (0.3 - 0.5) |
| 1983 | 4.5 (1.2 - 11.1) | 1.7 (1.1 - 2.4) | 0.9 (0.6 - 1.2) | 0.4 (0.2 - 0.5) |
| 1984 | 3.7 (0.6 - 10.0) | 0.9 (0.6 - 1.4) | 0.7 (0.4 - 0.9) | 0.0 (0.0 - 0.0) |
| 1985 | 2.9 (0.0 - 8.5) | 0.0 (0.0 - 0.0) | 0.0 (0.0 - 0.0) | 0.0 (0.0 - 0.0) |
| 1986 | 2.8 (0.3 - 7.8) | 0.3 (0.2 - 0.4) | 0.3 (0.2 - 0.4) | 0.3 (0.2 - 0.4) |
| 1987 | 5.2 (2.2 - 10.6) | 2.7 (1.8 - 3.3) | 2.6 (1.7 - 3.2) | 1.9 (1.2 - 2.4) |
| 1988 | 4.9 (2.1 - 10.1) | 2.7 (1.8 - 3.6) | 2.2 (1.5 - 2.9) | 1.2 (0.8 - 1.6) |
| 1989 | 3.9 (1.2 - 9.4) | 1.5 (1.0 - 2.0) | 1.2 (0.8 - 1.6) | 0.5 (0.3 - 0.7) |
| 1990 | 2.9 (0.5 - 7.8) | 0.7 (0.4 - 1.0) | 0.3 (0.2 - 0.4) | 0.0 (0.0 - 0.0) |
| 1991 | 3.4 (1.1 - 8.4) | 1.1 (0.8 - 1.5) | 0.9 (0.5 - 1.1) | 0.7 (0.4 - 0.9) |
| 1992 | 1.9 (0.1 - 6.5) | 0.1 (0.1 - 0.2) | 0.0 (0.0 - 0.0) | 0.0 (0.0 - 0.0) |
| 1993 | 2.7 (0.8 - 7.2) | 0.9 (0.6 - 1.2) | 0.6 (0.4 - 0.8) | 0.5 (0.3 - 0.6) |
| 1994 | 2.4 (0.8 - 6.1) | 1.0 (0.7 - 1.4) | 0.8 (0.6 - 1.1) | 0.3 (0.2 - 0.4) |
| 1995 | 2.9 (1.3 - 6.9) | 1.5 (1.0 - 2.0) | 1.2 (0.8 - 1.6) | 0.8 (0.5 - 1.2) |
| 1996 | 1.9 (0.5 - 5.8) | 0.5 (0.3 - 0.7) | 0.3 (0.2 - 0.4) | 0.2 (0.1 - 0.3) |
| 1997 | 1.7 (0.5 - 5.0) | 0.6 (0.3 - 1.0) | 0.1 (0.1 - 0.2) | 0.0 (0.0 - 0.0) |
| 1998 | 2.1 (0.9 - 5.9) | 0.9 (0.6 - 1.3) | 0.6 (0.4 - 0.9) | 0.3 (0.2 - 0.5) |
| 1999 | 2.7 (1.1 - 6.6) | 0.9 (0.5 - 1.4) | 0.2 (0.1 - 0.4) | 0.2 (0.1 - 0.2) |
| 2000 | 1.4 (0.4 - 4.2) | 0.1 (0.1 - 0.2) | 0.0 (0.0 - 0.0) | 0.0 (0.0 - 0.0) |
| 2001 | 2.4 (1.2 - 5.6) | 1.1 (0.7 - 1.8) | 0.7 (0.4 - 1.1) | 0.2 (0.1 - 0.4) |
| 2002 | 2.9 (1.6 - 5.7) | 1.7 (0.8 - 2.7) | 1.4 (0.5 - 2.4) | 0.9 (0.2 - 1.7) |
| 2003 | 2.4 (1.1 - 5.3) | 0.8 (0.4 - 1.3) | 0.5 (0.2 - 0.9) | 0.1 (0.0 - 0.3) |
| 2004 | 1.5 (0.6 - 4.1) | 0.2 (0.1 - 0.3) | 0.1 (0.0 - 0.1) | 0.0 (0.0 - 0.0) |
| 2005 | 2.7 (1.2 - 5.6) | 0.7 (0.3 - 1.2) | 0.3 (0.1 - 0.7) | 0.2 (0.0 - 0.5) |
| 2006 | 2.4 (1.1 - 5.3) | 0.6 (0.2 - 1.1) | 0.3 (0.0 - 0.6) | 0.1 (0.0 - 0.3) |
| 2007 | 1.8 (0.7 - 4.5) | 0.6 (0.2 - 1.0) | 0.2 (0.1 - 0.4) | 0.0 (0.0 - 0.0) |
| 2008 | 1.9 (0.6 - 5.1) | 0.1 (0.0 - 0.1) | 0.0 (0.0 - 0.0) | 0.0 (0.0 - 0.0) |
| 2009 | 1.9 (0.8 - 4.6) | 0.4 (0.1 - 0.7) | 0.1 (0.0 - 0.3) | 0.0 (0.0 - 0.1) |
| 2010 | 2.7 (1.2 - 6.1) | 0.9 (0.3 - 2.0) | 0.2 (0.0 - 0.9) | 0.0 (0.0 - 0.7) |
| 2011 | 2.4 (0.9 - 6.0) | 0.4 (0.1 - 0.8) | 0.0 (0.0 - 0.1) | 0.0 (0.0 - 0.0) |
| 2012 | 3.2 (1.3 - 7.3) | 0.4 (0.1 - 1.0) | 0.2 (0.0 - 0.6) | 0.0 (0.0 - 0.2) |
| 2013 | 2.2 (0.9 - 5.7) | 0.3 (0.1 - 0.9) | 0.0 (0.0 - 0.4) | 0.0 (0.0 - 0.1) |
| 2014 | 2.6 (0.9 - 6.6) | 0.1 (0.0 - 0.3) | 0.0 (0.0 - 0.2) | 0.0 (0.0 - 0.1) |
| 2015 | 3.1 (1.1 - 7.5) | 0.3 (0.1 - 0.7) | 0.1 (0.0 - 0.3) | 0.0 (0.0 - 0.1) |
| 2016 | 3.3 (1.1 - 7.7) | 0.1 (0.0 - 0.4) | 0.0 (0.0 - 0.2) | 0.0 (0.0 - 0.0) |
| 2017 | 2.3 (0.7 - 6.2) | 0.1 (0.0 - 0.2) | 0.0 (0.0 - 0.0) | 0.0 (0.0 - 0.0) |
| 2018 | 3.8 (1.2 - 8.0) | 0.5 (0.0 - 1.2) | 0.0 (0.0 - 0.3) | 0.0 (0.0 - 0.1) |

# Second-stage functional regression through boosting

The second-stage functional regression model is fitted using FDboost (Brockhaus and Ruegamer, 2016) which applies the gradient boosting ideas developed by Bühlmann and Hothorn (2007).

The model consists of eight base learners, one for each of the climate indices and one for the time component. Each of the base learners is represented by P-splines (Eilers and Marx, 1996) with 10 knots, which are penalized in order to obtain a smooth component with an equivalent of four degrees of freedom. At each iteration of the boosting algorithm, a single base learner is fit to the current residuals and added to the model with a weight corresponding to the step length of the algorithm chosen here as 0.01 which corresponds to small steps in order to give more opportunities to the algorithm to include climate indices. This base learner is chosen as the one minimizing the residual sum of squares. The algorithm stops after a predetermined number of iterations.

The optimal number of iterations is chosen between 10 and 500 by 10-fold cross-validation for each model. The chosen number of iterations is shown in Table S3.

Table S3: Optimal number of iterations of the boosting algorithm for each model.

| **Percentile** | **Montreal** | **Quebec** |
| --- | --- | --- |
| MMT | 69 | 131 |
| 95^th^ | 10 | 76 |
| 97.5^th^ | 10 | 90 |
| 99^th^ | 10 | 32 |

# Sensitivity analysis

Tables S4 and S5 show cross-validated R^2^ and selected variables of the second-stage models when average temperature is included. Although the cross-validated R^2^ is increased compared to the main model, especially in Montreal, results are consistent between the models. AMO is still selected in each model despite the presence of temperature.

Table S4: Cross-validated R^2^ (standard deviation) expressed in percentage for models including temperature.

| **Percentile defining heat** | **Montréal** | **Québec** |
| --- | --- | --- |
| MMT | 62.5 (5.0) | 72.5 (7.2) |
| 95^th^ | 15.5 (1.6) | 41.0 (3.4) |
| 97.5^th^ | 7.3 (0.7) | 44.9 (5.3) |
| 99^th^ | 2.5 (0.4) | 22.1 (3.3) |

Table S5: Selected variables for models including temperature. Variable ‘Time’ refers to the $s\left( i \right)$ term in Equation (1) of the main manuscript.

| **Percentile defining heat** | **Montréal** | **Québec** |
| --- | --- | --- |
| MMT | AMO, NAO, Time, Temperature | AMO, NAO, PDO, Time, Temperature |
| 95^th^ | AMO, Temperature | AMO, Time, Temperature |
| 97.5^th^ | Temperature | AMO, PDO, SOI, Time, Temperature |
| 99^th^ | AMO, Temperature | AMO, Time |

# References

Assani, A.A., Maloney-Dumont, V., Pothier-Champagne, A., Kinnard, C., Quéssy, J.-F., 2019. Comparison of the temporal variability of summer temperature and rainfall as it relates to climate indices in southern Quebec (Canada). Theor. Appl. Climatol. 137, 2425–2435. https://doi.org/10.1007/s00704-018-2750-8

Brockhaus, S., Ruegamer, D., 2016. FDboost: Boosting Functional Regression Models.

Bühlmann, P., Hothorn, T., 2007. Boosting Algorithms: Regularization, Prediction and Model Fitting. Stat. Sci. 22, 477–505.

Eilers, P.H.C., Marx, B.D., 1996. Flexible Smoothing with $B$-splines and Penalties. Stat. Sci. 11, 89–102.

Gershunov, A., 1998. ENSO Influence on Intraseasonal Extreme Rainfall and Temperature Frequencies in the Contiguous United States: Implications for Long-Range Predictability. J. Clim. 11, 3192–3203. https://doi.org/10.1175/1520-0442(1998)011<3192:EIOIER>2.0.CO;2

Ghosh, R., Müller, W.A., Baehr, J., Bader, J., 2017. Impact of observed North Atlantic multidecadal variations to European summer climate: a linear baroclinic response to surface heating. Clim. Dyn. 48, 3547–3563. https://doi.org/10.1007/s00382-016-3283-4

Majeed, H., Coles, J.G., Moore, G.W.K., 2020. Influence of Atlantic and Pacific Sea Surface Temperatures on Heat-Related Mortality in the United States. Geohealth 4, UNSP e2019GH000220. https://doi.org/10.1029/2019GH000220

Mantua, N.J., Hare, S.R., 2002. The Pacific Decadal Oscillation. J. Oceanogr. 58, 35–44. https://doi.org/10.1023/A:1015820616384

McCabe, G.J., Palecki, M.A., Betancourt, J.L., 2004. Pacific and Atlantic Ocean influences on multidecadal drought frequency in the United States. Proc. Natl. Acad. Sci. 101, 4136–4141. https://doi.org/10.1073/pnas.0306738101

Seager, R., Kushnir, Y., Nakamura, J., Ting, M., Naik, N., 2010. Northern Hemisphere winter snow anomalies: ENSO, NAO and the winter of 2009/10. Geophys. Res. Lett. 37. https://doi.org/10.1029/2010GL043830

Thompson, D.W.J., Wallace, J.M., 1998. The Arctic oscillation signature in the wintertime geopotential height and temperature fields. Geophys. Res. Lett. 25, 1297–1300. https://doi.org/10.1029/98GL00950
